# Exploring Medical Student and Staff Perspectives on Clinical Placements: Factors Influencing Attendance and Engagement

**DOI:** 10.7759/cureus.93336

**Published:** 2025-09-27

**Authors:** Hannah J Sadler, Aneeta A John, Sophie Thomas, Laura Sevenoaks, Lauren J Medwell, Anila Viepadan

**Affiliations:** 1 Medical Education, Great Western Hospital National Health Service (NHS) Trust, Swindon, GBR

**Keywords:** attendance rates, experiences of medical student placements, healthcare staff perspectives, improving clinical placements, medical student perspectives, medical student placements

## Abstract

Background

Medical students are expected to spend a significant amount of time on clinical placement as part of their training. However, attendance rates at placements are often low, and engagement is inadequate. This has a negative impact on medical student learning and wastes NHS and university resources.

Aims

This study aims to gain insight into both medical student and clinical staff experiences of placements to identify reasons behind low attendance and possible improvements.

Methods

This study utilised an exploratory mixed-methods design using questionnaires to collect both qualitative and quantitative data including Likert scales and multiple-choice and ranking questions from staff and student respondents at one large NHS trust, UK. Participants included staff working in clinical areas in which students are based as well as medical students on placement at the trust from three different UK universities. Data were analysed using R (R Foundation for Statistical Computing, Vienna, Austria), and between-group comparisons were performed for four thematically matched items using the Mann-Whitney U test. Statistical significance was set at p < 0.05. Qualitative analysis was undertaken using inductive thematic analysis by multiple researchers to ensure coding consistency.

Results

We received 87 responses (46 from students and 41 from staff). Quantitative data revealed that 63.0% of students had been turned away from clinical placement and 30.0% did not feel part of the team when attending. Students rated the availability of valuable learning opportunities lower (median = 4) compared with staff (median = 5), with a significant difference (U = 561.5, p < 0.001, r = -0.40). Conversely, students perceived that there were more medical students than opportunities (median = 3) compared with staff (median = 2), also significantly different (U = 1328.5, p < 0.001, r = 0.41). No significant difference was observed regarding whether the number of students was appropriate (U = 994.0, p = 0.64, r = 0.05). Similarly, perceptions of student preparedness did not differ significantly (U = 1,122.5, p = 0.087, r = 0.19), although staff ratings trended lower. Qualitative analysis identified overlapping themes from students and staff, including organisation and integration into the team, alongside student-specific themes of workplace-based teaching, staff awareness, feeling welcome, and responsibility, and staff-specific themes of communication and cultural attitudes towards education.

Conclusion

This study highlighted several key reasons behind poor engagement and attendance of medical students in clinical placements. These include communication barriers, poor organisation, lack of team integration, and overcrowding. These can be overcome with simple, practical solutions such as pre-allocating students to staff, sharing student timetables with clinical staff, careful timetabling to avoid student clashes, and improved availability of teaching in the clinical environment.

## Introduction

Clinical placements are an essential component of the UK's medical school curriculum as laid out by the General Medical Council [1]. The clinical environment provides opportunities for students to apply knowledge and skills to practice with meaning and purpose. Workplace learning underpins many theoretical frameworks within medical education. Kolb’s Experiential Learning Theory outlines how learning occurs through a cycle of experience, reflection, conceptualisation, and application within the workplace [2]. Lave and Wenger’s Situated Learning Theory similarly emphasises that workplace learning enables students to become increasingly autonomous through legitimate peripheral participation [3]. Clinical experiences with patients and staff are irreplaceable in these processes.

Despite the clear importance of clinical placements, limited data exist on the attendance rates of medical students in clinical placements. However, evidence of declining attendance rates to classroom-based sessions [4] is likely to be mirrored in clinical placements, as suggested by anecdotal accounts of poor attendance from educators and clinicians in the field of medical education. A recent audit at our trust, a large NHS district general hospital, showed that attendance rates of medical students to clinical placement were just 50% across three UK medical schools over a period of five weeks.

Increasing student numbers means that demand for clinical spaces for medical students is growing, putting greater pressure on organisations to increase capacity [5]. Universities often spend a large proportion of their budget to provide placement experiences for their students [6]. Low attendance rates risk wasting valuable resources, including financial costs of placement, clinical spaces that could be utilised by other healthcare students, and staff time dedicated to organising and allocating placements.

Attendance to clinical placements has been shown to directly correlate with academic performance in medical school and preparedness for work as a doctor [7-10]. Clinical placements provide the opportunity to develop essential skills required to care for patients, such as communication, professionalism, and multidisciplinary team working [8,10]. In other healthcare professions such as nursing, negative experiences whilst on placement as a student have been identified as a major contributor to student drop-out rates [11,12]. It is, therefore, vital that universities and hospitals work together to improve the attendance rates and experience of clinical placements.

Poor attendance during medical school has been highlighted as a key issue previously; however, most literature focuses on classroom-based attendance [13-15]. Studies that have looked at experiences on clinical placement fail to get a broad range of views due to small sample sizes and limited participants [16-18]. This exploratory study is the first to investigate the experiences of medical student placements across multiple universities, incorporating perspectives from both staff and students. This study aims to explore the views on clinical placements, including factors influencing attendance and engagement, as well as suggestions for improvement. By employing a mixed-methods approach, the study will quantify issues such as overcrowding and team integration whilst gaining deeper insights into the lived experiences of students and staff.

## Materials and methods

Study structure 

This study employed a mixed-methods approach to achieve a comprehensive understanding of the factors influencing placement experiences and attendance. This method enabled the collection of quantitative data on issues such as overcrowding and team integration whilst also providing qualitative insights into the context and meaning behind these findings. Triangulating the quantitative and qualitative results enhanced the validity and credibility of the study's conclusions.

Sampling 

The study took place in Great Western Hospital, a large NHS district general hospital that organises clinical placement for medical students from three separate UK universities. Participants involved both medical students on placement and staff who work in clinical areas in which medical students are allocated. Inclusion criteria included any member of staff within the trust working in clinical areas that accommodate medical students and medical students on placement in the trust at the time.

Study participation was advertised via email and poster displays to all students on placement and all trust staff working in clinical areas. Whilst this approach was considered the most practical method of recruitment, it is important to acknowledge that it may have introduced a degree of self-selection bias.

Data collection 

Survey data were collected separately from medical students and staff using two different questionnaires (Appendices A, B). The questionnaires were developed by resident doctors with experience in both clinical work and medical education, and they underwent peer review prior to distribution to ensure relevance and appropriateness. However, the questionnaires were not formally validated, and this reduces the validity of the data. Students completed six items relating to their perceptions of ward-based learning opportunities, preparedness, and integration into the clinical team. Staff completed eight items focusing on their ability to support medical students alongside their own responsibilities, and their perceptions of student engagement, professionalism, and preparedness. All items were scored using a five-point Likert scale (1 = strongly disagree to 5 = strongly agree). In addition, both study groups were asked multiple-choice questions, and students were additionally asked a ranking question. Qualitative data were collected using open-ended free-text questions.

Data analysis 

The Likert data were analysed using R (version 4.4.1, R Foundation for Statistical Computing, Vienna, Austria). Given the ordinal nature of Likert data and non-normal distributions, non-parametric tests were employed. Descriptive statistics are reported as medians and interquartile ranges (IQR). Within-group analysis summarised each item separately for students and staff. Between-group comparisons were performed for four thematically matched items (valuable learning opportunities, number of students vs. learning opportunities, appropriateness of student numbers, and preparedness for placements) using the Mann-Whitney U test. Effect sizes were calculated using the rank-biserial correlation (r), with 0.1, 0.3, and 0.5 interpreted as small, moderate, and large effects, respectively. Statistical significance was set at p < 0.05. Estimated sample size calculations were performed using G*Power (Heinrich-Heine-Universität Düsseldorf, Düsseldorf, Germany), with rank-biserial correlations from the Mann-Whitney U tests converted to Cohen’s d and entered into a two-tailed t-test family model (α = 0.05, power = 0.80), indicating that approximately 40 participants per group would be required to detect the observed moderate effects.

Qualitative data were analysed using inductive thematic analysis within a constructivist framework. This approach meant that themes were developed directly from participants’ accounts, whilst recognising that their experiences and perspectives are shaped by the social and institutional context of medical education. Analysis was conducted using Braun and Clarke’s six-step process for thematic analysis to identify themes and subthemes [19]. Coding was undertaken by clinicians involved in medical education, and we recognise that the backgrounds and experiences of the coders will inevitably have influenced the qualitative data collection and interpretation. The researchers' roles as teachers, curriculum designers, and assessors within medical education provide insider perspectives that help us understand the nuances of participants’ experiences. At the same time, this method risks introducing bias, particularly if assumptions are made based on personal experiences. Initial codes were generated manually, from which themes and subthemes emerged. Coding consistency was ensured using detailed inclusion and exclusion criteria for each code; additionally, each data set was coded independently by multiple trained researchers, and convergence was assessed qualitatively through discussion. Themes were defined as broad overarching topics of frequently mentioned ideas, with subthemes being specific ideas that were often reported.

Ethics approval 

This study received ethical approval from the Swindon Medical Education Research Committee in November 2024, Ethics approval code AJ1124. All participants signed informed consent forms prior to engagement in the study. Data were collected and stored anonymously using secure, encrypted databases and destroyed following analysis.

## Results

Respondent demographics 

We had 87 responses to our survey: 46 from medical students and 41 from health professional staff. The response rate was estimated to be around 23.0% for students and 5.0% for staff. The student responses came from three different medical schools across all clinical year groups (Appendix C). The majority of respondents to the staff survey were resident doctors at 78.0% (32). Consultant doctors made up 14.6% (6) of responses, 2.4% (1) each from nursing staff, administrative staff, and other staff. The responses covered a wide range of clinical areas including medical, surgical, women’s health, paediatric, and acute services (see Appendix D). 

Quantitative results 

*Student Numbers on Placements* 

To explore whether increasing student numbers may contribute to overcrowding, two questions focused on the number of students present and whether students were being turned away. The majority of both students and staff (73.9% (34) and 85.4% (35), respectively) reported that, on average, between one and two additional students are present in a clinical area at one time; however, a significant minority, 13.0% (6) of students and 14.6% (6) of staff, reported this to be between three to four students.

Regarding being turned away from wards, this had happened at least once to 63.0% (29) of students, with 10.9% (5) reporting this had occurred more than four times. The staff respondents did not report awareness of students being turned away, with 53.7% (22) disagreeing; however, a significant minority, 24.4% (10), agreed that students had been turned away, and 22.0% (9) were unsure.

Student Experiences of Clinical Placements 

**Figure 1:**
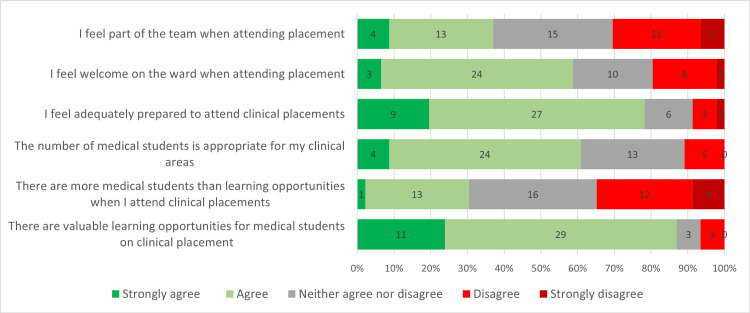
Graph displaying the distribution of student responses to various questions on a Likert scale.

The responses to Likert scales used to assess the views of students on clinical placements are shown in Figure 1. Student responses demonstrated consistently positive perceptions of placements (Table 1). Students reported that valuable learning opportunities were present (median = 4, IQR = 0), that the number of medical students was appropriate (median = 4, IQR = 1), and that they felt adequately prepared for placements (median = 4, IQR = 0). Students also reported feeling welcome (median = 4, IQR = 1) and part of the team (median = 3-4, IQR = 1). The only item with more variability was the perception that there were more students than available opportunities (median = 3, IQR = 2).

**Table 1:** Student responses to Likert scales (n, median, IQR). IQR: interquartile range

Question	n	Median	IQR
There are valuable learning opportunities for medical students on clinical placement	46	4	0
There are more medical students than learning opportunities when I attend clinical placements	46	3	2
The number of medical students is appropriate for my clinical areas	46	4	1
I feel adequately prepared to attend clinical placements	46	4	0
I feel welcome on the ward when attending placement	46	4	1
I feel part of the team when attending placement	46	3	2

**Figure 2:**
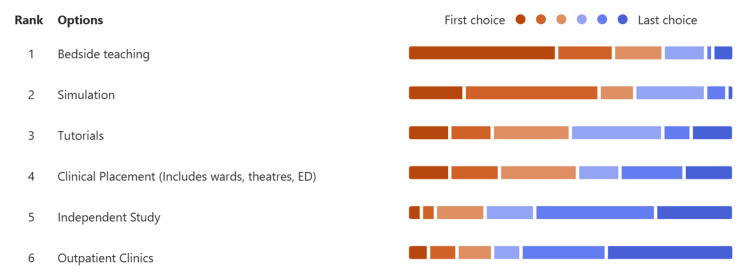
Graph displaying the students' ranking of best to worst learning opportunities on placement. ED: emergency department

Students ranked different types of learning opportunities on placement from most to least valuable, as seen in Figure 2. Clinical placements ranked fourth out of six, with 13.0% (6) ranking it first place and 15.2% (7) ranking it last. Bedside teaching ranked first with 45.7% (21) of students ranking this as the most valuable experience for learning.

*Staff Experiences of Medical Student Clinical Placements * 

**Figure 3:**
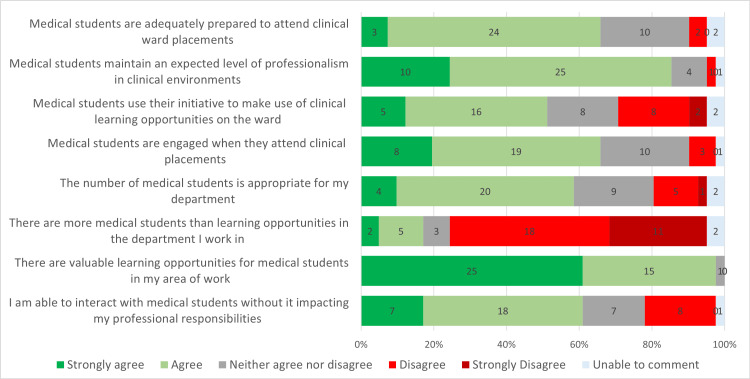
Graph displaying the distribution of staff responses to various questions on a Likert scale.

Likert scale responses used to assess the staff experiences of student clinical placements are shown in Figure 3. Staff also rated their experience of teaching students positively (Table 2). They strongly endorsed that valuable learning opportunities existed within their areas (median = 5, IQR = 1) and that medical students generally maintained professionalism (median = 4, IQR = 0-1). Staff responses suggested that student preparedness (median = 4, IQR = 0-1) and engagement (median = 4, IQR = 1) were appropriate, whilst some variability was observed in ratings of student initiative (median = 3-4, IQR = 1). Staff tended to perceive that the number of students sometimes exceeded opportunities (median = 2, IQR = 1).

**Table 2:** Staff responses to Likert scales (n, median, IQR). IQR: interquartile range

Question	n	Median	IQR
I am able to interact with medical students without it impacting my professional responsibilities	41	4	1
There are valuable learning opportunities for medical students in my area of work	41	5	1
There are more medical students than learning opportunities in the department I work in	41	2	1
Number of students appropriate	41	4	1
The number of medical students is appropriate for my department	41	4	1
Medical students are engaged when they attend clinical placements	41	4	1
Medical students use their initiative to make use of clinical learning opportunities on the ward	41	4	1
Medical students maintain an expected level of professionalism in clinical environments	41	4	0
Medical students are adequately prepared to attend clinical ward placements	41	4	1

Student vs. Staff Comparisons 

**Figure 4:**
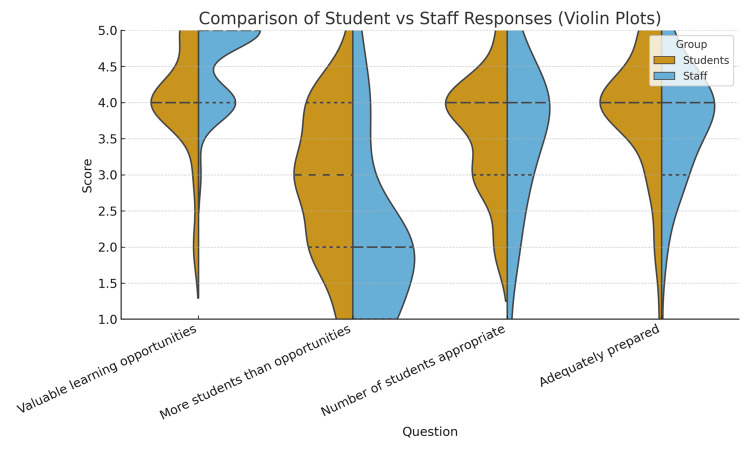
Violin plots showing the distribution of Likert ratings (1 = strongly disagree, 2 = disagree, 3 = neither agree nor disagree, 2 = agree, and 5 = strongly agree) for four matched items, comparing students and staff.

Between-group comparisons are presented in Table 3 and Figure 4. Students rated the availability of valuable learning opportunities lower (median = 4) compared with staff (median = 5), with a significant difference (U = 561.5, p < 0.001, r = -0.40), representing a moderate effect size. Conversely, students perceived that there were more medical students than opportunities (median = 3) compared with staff (median = 2), also significantly different (U = 1,328.5, p < 0.001, r = 0.41), another moderate effect. No significant difference was observed regarding whether the number of students was appropriate (U = 994.0, p = 0.64, r = 0.05). Similarly, perceptions of student preparedness did not differ significantly (U = 1,122.5, p = 0.087, r = 0.19), although staff ratings trended lower.

**Table 3:** Comparison of student and staff responses (medians, Mann-Whitney U tests, p-values, effect size).

Question	Student median	Staff median	Mann-Whitney U statistic	p-value	Effect size (r)
There are valuable learning opportunities for medical students on clinical placement	4	5	561.5	0.0003	-0.4
There are more medical students than learning opportunities when I attend clinical placements	3	2	1,328.5	0.0007	0.41
The number of medical students is appropriate for my clinical areas	4	4	994	0.6418	0.05
Medical students are adequately prepared to attend clinical ward placements	4	4	1,122.5	0.0867	0.19

Qualitative results 

Respondents were asked how ward placement experiences can be improved. Thematic analysis was conducted separately for the student respondents and staff respondents, which revealed nine themes in total, as shown below.

*Themes From Student Respondents* 

Five key themes were identified from thematic analysis of the student responses, as displayed in Table 4.

**Table 4:** Table displaying the themes and subthemes from student respondents.

Themes	Subthemes		Quotation
Workplace-based teaching	Motivators	Non-formal teaching	‘If someone was able to teach on wards’ Student #45
		Senior-led teaching	‘More consultant taught bedside teaching’ Student #35
		Clinical teaching fellow (CTF) led teaching	‘More teaching with CTFs on wards’ Student #37
		Bedside teaching	‘More bedside teaching’ Student #5
		Supporting students to develop confidence	‘Weekly sessions are very good for gaining confidence practically’ Student #33
	Barriers	Balancing clinical time with teaching	‘Seniors or consultants have not had the availability to teach while running a ward round’ Student #12
		Staff being too busy	‘They are understaffed’ Student #42
Staff awareness	Motivators	Staff expecting students	‘Would be helpful ... for wards to be expecting us’ Student #6
		Staff knowledge of curriculum requirements	‘Better knowledge of sign offs for students, as this is a big barrier to doing things like mini-CEXs as doctors don't realise what is involved so are reluctant to help’ Student #1
Organisation	Motivators	Increased flexibility	‘Having the flexibility to attend another ward’ Student #12
		Diversity of placement	‘Diversity of placement’ Student #24
		Length of placement	‘Some more time on 1 ward would allow us to integrate more’ Student #40
		Familiarity/continuity	‘Spending 2/3 consecutive days at the start of the week’ Student #3
		Assigned mentors	‘An assigned mentor/supervisor would be great’ Student #15
		Time for independent study	‘More scheduled time for independent study’ Student #41
		Paired placements	‘Being paired up with another student’ Student #25
				Barriers	Too many students	‘Fewer students’ Student #15
	Clashes with other student groups	‘Multiple students in one place’ Student #30		
	Feeling welcome	Motivators	Better induction to wards	‘More of an introduction to the ward’ Student #19
Willingness to teach					‘It’s been shocking how little enthusiasm has been shown towards medical students during my time here’ Student #2
Feeling part of a team			‘Feel more integrated in team’ Student #18
Barriers			Feeling unwelcome	‘(I) want to feel more welcome’ Student #7
		Feeling ignored	‘Drs tend to talk over you’ Student #46
		Feeling like a burden	‘Don’t want to burden healthcare team’ Student #42
				Being given responsibility	Motivators	More clinical skills exposure	‘(I want) staff in clinics or ward doctors to be open for us to get more involved in ward rounds or clinical skills’ Student #44
Being assigned jobs		‘To feel useful on the wards! Eg. Asked to do bloods, or to scribe on the round’ Student #46			
Confidence to see patients	‘Be more comfortable to see patients’ Student #19				
Opportunities to see interesting clinical signs	‘Have juniors flag good patients to examine’ Student #18				

Workplace-based teaching: Students often reported wanting more teaching when they are present in clinical areas from near-peer and senior staff such as bedside or clinical skills teaching. There was a common feeling that staff were often too busy or uninterested to provide any teaching for students. 

‘Supplemented with more teaching I think we could be very successful on the wards. Independent study alone sometimes makes it difficult because it’s hard to know what you should know before you attend. More teaching would be beneficial’. Student #28

Staff awareness: Students frequently stated that staff were often not expecting them in clinical areas, having a detrimental effect on placement experiences. Students also felt that staff often had no knowledge of university curriculum requirements such as portfolio sign-offs, making it difficult for students to achieve these. 

‘Ensure the ward or other clinical placement team is expecting us, have provisions for us, and are actually willing to help. It’s been shocking how little enthusiasm has been shown towards medical students during my time here’. Student #2 

Organisation: Several respondents reported that having an assigned member of staff that they could turn to in clinical areas would be important for improving placement experience. It was frequently mentioned that too many students in clinical areas led to missing out on learning opportunities. Several students also wanted increased continuity with longer placements or consecutive time in an area. 

‘There were occasions where the consultant felt there were too many students and said some had to leave. If the consultants know how many to expect and can reject beforehand, if necessary, rather than when people arrive this would be better’. Student #10

‘Make ward placements 3 weeks instead of 2. By the end of 2 weeks you are just starting to feel like a part of the team and leaving then can be quite off-putting. Especially since some weeks we have a lot more teaching than others I think some more time on 1 ward would allow us to integrate more and get used to the routine’. Student #40

Feeling welcome: Respondents frequently reported that they felt unwelcome in ward environments and were keen to feel more integrated into clinical teams.

‘It’s very difficult to integrate into a team when it’s obvious that they’re inconvenienced by you’. Student #36

‘(I) want to feel more welcome and part of the team’. Student #7

Being given responsibility: Students report they are keen to get more involved with ward tasks such as on the ward round, clinical skills, or clerking. Having more independence was also reported as something that would improve placements. 

‘To feel useful on the wards! Eg. Asked to do bloods, or to scribe on the round, or to look up results on the computer’. Student #46

‘More independence for clinical skills’. Student #21

*Themes From Staff Respondents* 

Four key themes were identified from the thematic analysis of the staff responses, as displayed in Table 5.

**Table 5:** Table displaying the themes and subthemes from staff respondents.

Themes	Subthemes		Quotation
Organisation	Motivators	Change in current teaching structure	‘Placement to be structured with learning objectives’ Staff #2
				Better organisation	‘Avoiding morning teaching sessions as the students miss out on ward round’ Staff #41
		More staff needed to teach	‘Better ward staffing’ Staff #8
		More IT access for students	‘Please give them (students) IT log ins’ Staff #18
		Continuity	‘Give students consistent placements so they are able to embed themselves in the team over several days’ Staff #35
				Fewer curriculum sign-offs	‘Minimize amount of “checklist” style portfolio/log-book items they have to do’ Staff #15
		Specific goals/aims for students on wards	‘Encourage medical students to come with aims’ Staff #17
		Barriers	Too many students	‘Sometimes we have 6-7 medical students in SAU, and there aren’t enough learning opportunities for them, so we do end up turning people away’ Staff #28
		Communication	Motivators	Communication between clinical teaching fellows and ward staff	‘It could be improved if we had a roster and awareness who was coming and when’ Staff #26
	Awareness of available opportunities			‘Consultants could have a timetable of educational opportunities for them to attend’ Staff #21
Cultural attitudes towards education	Motivators	Better staff engagement/willingness to teach	‘By ensuring Consultants get actively involved in their teaching ward rounds. It shouldn't be optional but an expectation’ Staff #34
		Better student engagement/enthusiasm	‘If they are willing to engage with some jobs e.g. bloods, facilitating a patient going for an investigation etc, that will free up staff's time to then dedicate to teaching them’ Staff #35
		Incentives for staff	‘We are not paid for, and there is no additional time in our job plans, for teaching, so to make time they need to help us’ Staff #35
	Barriers	Staff too busy to teach	‘The ward can be very busy and we need to put patient treatment first’ Staff #31
		Lack of confidence in basics prior to placement	‘If they (students) are confident in the basics they can be more helpful’ Staff #29
Feeling part of a team	Motivators	Allocated doctors for students	‘Linking them to specific doctors’ Staff #11
				Student autonomy	‘Better if given more independent tasks’ Staff #13

Organisation: Staff reported wanting placements to be structured in line with learning objectives and aims. Many respondents also mentioned understaffing as a key issue with clinical demands making teaching difficult. It was also highlighted that morning placements yield greater learning experiences and fewer students would improve the experience. 

‘Coming in the morning for ward rounds helps as this is when we can do more teaching. In the afternoons there's a lot of admin tasks which are not helpful for their learning for example sitting watching us make phone calls or write discharge letters isn't very helpful. If they had IT logons it would be better so they could practice some clinical tasks. We also don't know what their learning objectives are, this could help me target the teaching’. Staff #31

‘Linking them to specific doctors that they can shadow. Allowing some time to that doctor to teach/involved medical students in ward proceedings (for example, that doctor could be given 30-45 mins where he/she would be only teaching the student)’. Staff #11

Communication: Staff respondents reported that they wanted to be better informed prior to medical students arriving in clinical areas as well as better communication with students once on the wards. 

‘More transparency on who and how many are attending and for how long. Medical students sometimes say they are with you for only a few days, when in fact it is the entire week, allowing them to engage or disengage more easily’. Staff #21

‘Clear bilateral (students and clinicians) expectation of being on the ward rather than formal teaching breaking the experience up - the relevant team should be told that they will have medical students present’. Staff #38

Cultural attitude towards education: Staff reported that engagement was an issue both from staff and students, and many feel that if students show more willingness to get involved and help with jobs, then their experiences will be better. They also felt staff were often too busy to help students and needed incentives to teach.

‘Students also do not seem to understand that they need to contribute to the team. If they are willing to engage with some jobs e.g. bloods, facilitating a patient going for an investigation etc, that will free up staff's time to then dedicate to teaching them. We are not paid for, and there is no additional time in our job plans, for teaching, so to make time they need to help us’. Staff #35

Feeling part of a team: Staff respondents felt placements would be improved if students were allocated to a named staff member in advance to help integrate them into the clinical teams. 

‘It works well in ED because the students are allocated to a resident doctor in advance. The resident doctor is then prepared for this ahead of time’. Staff #36

‘I think if they are confident in the basics they can be more helpful, and that leads to better learning opportunities. For example, if they can take and document routine obs, take bloods, insert cannulas, then they start to work as part of the team. If all they are doing is taking histories and observing then the opportunities start to become rapidly saturated’. Staff #29 

## Discussion

This study aimed to explore the views of both medical students and clinical staff on factors influencing attendance and engagement in clinical placement. The overall goal of this study was to identify reasons behind poor attendance and non-engagement and highlight possible solutions to this issue. Our mixed-methods approach gained a large number of responses from medical students and staff members, providing invaluable insights into the experiences, opportunities, and challenges of medical student placements.

Summary of key findings

Our results from both quantitative and qualitative data identified that students and staff alike find clinical placements valuable opportunities for learning (87.0% (40) of students and 97.6% (40) of staff agreed), which is consistent with previous studies [7,8,20]. However, clinical placements are ranked fourth by students in terms of valued learning opportunities after bedside teaching, simulation, and tutorials, highlighting the need for improvement. 

Quantitative results revealed that being turned away from clinical areas is a significant issue for medical students. Over 63% (29) of students reported that they have experienced this, and 24.4% (10) of staff members acknowledged that their areas are turning students away. This was also highlighted within the qualitative results, with both staff and students reporting that too many students in clinical areas has resulted in students being turned away. The qualitative results also suggested that both staff and students felt that having fewer students in clinical areas would improve experiences on clinical placement. This suggests that overcrowding is negatively impacting placement experiences, an issue that is likely to get worse with increasing numbers of medical students. 

Another major barrier to engagement and attendance emphasised in both quantitative and qualitative results was the lack of integration into the clinical team, a finding supported by previous literature [10,21]. Although most felt part of a team (median 3-4), a significant proportion, 30.0% (14), reported feeling excluded from clinical teams. This was further demonstrated in the qualitative data with themes of ‘feeling welcome’ and ‘feeling part of a team’ from the student and staff results, respectively. The common occurrence of these themes highlights an area of real concern about the attitude towards medical students on placement. This inability to become ingrained within a team appears to be the result of several factors including incivility from staff, lack of student confidence, and lack of proactivity from students. Feeling ignored or unwelcome came up commonly in the student data; incivility such as this is a well-documented issue experienced by medical staff around the world [21,22]. 

Themes that emerged from qualitative results included the need for better organisation, and both students and staff suggested changes such as improved continuity and altered timetabling structure. Students were especially keen for more formal teaching whilst in clinical areas such as bedside teaching and Socratic questioning as well as increased opportunities for clinical skills practice. Staff respondents emphasised the importance of knowing students’ learning objectives and goals for their time in clinical areas. 

Communication was identified as a key area for improvement with both students and staff wanting more knowledge of who was attending which areas and when. Improving communication between the staff members who arrange placements and staff in clinical areas where students attend could enable staff to prepare for students, arrange appropriate opportunities, and prevent hostility when students turn up unexpectedly in clinical areas.

Practical implications of findings

The findings of this study highlight several actionable areas laid out below for the improvement of clinical placements and the increase of attendance and engagement rates.

Changing the Structure of Placement 

Changing the structure of placements to ensure ward time is scheduled in the mornings with classroom teaching reserved for afternoons would help integrate students into the team, be assigned roles, and experience senior-led teaching on ward rounds. This would allow students to be introduced during morning board rounds and foster a sense of belonging, something that has been shown to be effective in increasing attendance in other studies [23-25]. Furthermore, incorporating student-selected modules into clinical placements has been shown to benefit student perceptions of clinical integration within teams [26]. Giving students more freedom to choose which clinical areas they attend could help with engagement and attendance. However, this would require significant organisational planning and would have to be balanced with ensuring adequate exposure to a variety of areas to meet curriculum requirements. 

*Pre-defined Learning Objectives* 

Providing staff with clear, pre-defined learning objectives for students whilst on clinical placements may be a simple but effective action as shown by previous studies [16,25]. With oversight from the university to ensure appropriateness, encouraging students to create their own personal learning objectives will lead to increased satisfaction with placements [27,28]. This does need to be approached with caution - too much emphasis on meeting defined learning objectives may lead students to be hyper-focused on specific tasks and, therefore, miss out on other learning opportunities [29]. This can lead to staff and patient frustration; therefore, learning objectives would need to be flexible, broad, and without a need for sign-offs. An efficient means to provide these learning outcomes would need to be considered, for example, sending them to staff via email. This may be difficult to incorporate into regular practice due to the number of different staff who work on each ward, as well as the many different cohorts of students that can arrive on a ward for placement, each with their own different learning outcomes.

Assigning Students to Staff Members 

Thirdly, preassigning students to individual members of staff was an idea strongly supported by staff and students within our study. This ensures staff are expecting students and have adequate time to prepare for this whilst ensuring accountability and acceptance of that student. Near-peer clinical mentors for students can improve confidence and preparedness for work and encourage specialty interests, and so, effort should be made to allocate students to junior staff members [30]. This strategy could help implement the previous change by providing a channel of communication to share students' individual learning outcomes with staff members.

*Workplace Teaching* 

Our study highlighted that students wanted more teaching in the clinical environment. This can often be difficult to achieve in practice due to clinical responsibilities being prioritised, with chronic understaffing leaving no time to teach. However, involving clinical staff members in teaching wherever possible would result in improved student satisfaction. Immersing staff members in teaching, especially those who are more senior, and allowing them to be involved in designing the teaching schedule could also increase staff involvement [31], therefore leading to more opportunities for students.

Educating staff with the necessary knowledge and resources needed to teach in the work environment is the first step to this. This could involve information posters or educational sessions that provide staff with basic introductions to clinical teaching and practical tips on common workplace teaching challenges such as facilitating effective bedside teaching or case-based discussions in a limited time. Educational activities could also involve familiarising staff with student requirements, which can help staff signpost students to relevant opportunities. Incentives may be required to engage staff such as certificates of teaching and awards for best clinical teachers.

Improved Communication

Improved communication between university staff and clinical staff is another area for change. Clinical staff should receive information well ahead of placements on when and where to expect students, with details such as student names (with or without photos), year group, length of placement, and learning needs such as workplace-based assessment requirements. Clinical staff should have an open channel of communication with university staff in case of issues with students, whether that be non-attendance, professionalism, or pastoral. Communication could also be improved between students and clinical staff, such as involving students in team messaging chats. However, this may not be feasible in practice due to strict data sharing and confidentiality rules.

Managing Increasing Numbers of Medical Students

Medical student numbers are increasing year on year, and many hospitals now provide placements to medical students from multiple different universities. Thought needs to be given to how to cater for this increasing number of medical students, particularly as our study highlights that medical students are being turned away when too many students are present. This can be managed with careful organisation and planning. Timetables should avoid clashes whenever possible, such as by utilising out-of-hour opportunities and structuring placements so that whilst one group of students participates in classroom-based teaching, the other is engaged in clinical placements. However, the lack of teaching spaces and educators will impact the feasibility of these changes. 

Learning within a work environment occurs best when a learner becomes fully immersed in the team [32], and these simple steps help to improve the integration of the student into the clinical team. Although an individual’s learning type can influence where and how a student learns best [33], effective learning in workplaces relies on acceptance of the learner by employees and active involvement in tasks [34,35]. In general, these solutions are not resource-intensive but require commitment and collaboration from both clinical staff, university staff, and medical students. Funding constraints, staff shortages, and institutional policies will all be challenges to overcome in order to bring these changes.

Study limitations 

Whilst this study provides valuable insights, there are several limitations that should be acknowledged. Firstly, the sample size, although adequate for analysis, was small, and the single site limits generalisability. The questionnaires were not formally validated, and this will reduce the validity of the conclusions. Additionally, the use of self-reported data also introduces the possibility of self-selection bias, especially with regard to questions surrounding being turned away from clinical spaces. Furthermore, the qualitative data were limited to one open-ended survey response from each respondent, which will not provide a full overview of participant experiences. Thematic analysis was conducted by those working within the field of medical education, whereby underlying assumptions and views could introduce bias into the results.

Recommendations for future research 

Whilst this study generates valuable recommendations, we recognise the challenges with implementing these practically, especially in clinical areas that take multiple cohorts of students from different year groups and universities. Future research could explore how to apply standardised approaches that overcome this issue by involving larger sample sizes across a greater number of sites. Additionally, exploring the role of administrative staff in organising placements may provide further insights into the challenges of implementing organisational changes and identify possible solutions. Longitudinal studies assessing the impact of specific interventions such as staff incentives or improved communication would provide valuable insights into sustainable actions.

## Conclusions

Our study highlights that there are several barriers contributing to the medical student’s clinical placement experience. Various factors include a lack of integration within clinical teams, poor communication and organisation of clinical placements, and too many students allocated to a given clinical area. This has implications for preparedness to work as a doctor and the ability to care for patients. Addressing this issue with the methods discussed above could result in improved attendance but will require commitment and engagement from all parties involved.

## Appendices

Appendix A

**Table 6:** Questions used in the student questionnaire.

Question number	Question
1	What is your current student cohort (including year group and university)?
2	On average, how many other medical students are also on the wards when you attend? (0, 1-2, 3-4, >4)
3	How many times have you been turned away from any clinical placement? (0, 1-2, 3-4, >4)
4	Please indicate the extent to which you agree with the following statements (strongly agree, agree, neither agree nor disagree, disagree, strongly disagree): • There are valuable learning opportunities for medical students on clinical placement. • There are more medical students than learning opportunities when I attend clinical placements. • I feel adequately prepared to attend clinical placements. • The number of medical students is appropriate for my clinical area. • I feel welcome on the ward when attending placement. • I feel part of the team when attending placement.
		5	Please rank the following in order of most valuable to your learning: ward placement, simulation, tutorials, bedside teaching, independent study.
		6	How can your ward placement experience be improved?

Appendix B

**Table 7:** Questions used in the staff questionnaire. ED: emergency department; ITU: intensive therapy unit

Question number	Question
1	What ward/department do you work in? (medical, surgical, ED/ITU, theatres, women's health, child's health, other)
2	What is your role? (resident doctor, consultant, nurse, allied health professional, administrative staff, other)
3	When medical students are present, how many medical students do you typically have on the ward at any one time? (1-2, 3-4, more than 4)
4	Has your clinical area ever turned students away? (yes, no, unsure)
5	Please indicate the extent to which you agree with the following statements (strongly agree, agree, neither agree nor disagree, disagree, strongly disagree, unable to comment): • I am able to interact with medical students without it impacting my professional responsibilities. • There are valuable learning opportunities for medical students in my area of work. • There are more medical students than learning opportunities in the department I work in. • The number of medical students is appropriate for my department. • Medical students are engaged when they attend clinical placements. • Medical students use the initiative to make use of clinical learning opportunities on the ward. • Medical students maintain an expected level of professionalism in clinical environments. • Medical students are adequately prepared to attend clinical ward placements.
		6	How can medical student ward placements be improved?

Appendix C

**Table 8:** Number of student responses by year of study.

Year of study	Number of responses
3	9
4	21
5	6
6	10
Total	46

Appendix D

**Table 9:** Number of staff responses by department and job role. ED: emergency department; ITU: intensive therapy unit

Department	Number of responses	Job title	Number of responses
Medical	16	Resident doctor	32
Surgical	7	Consultant	6
ED/ITU	5	Nurse	1
Theatres	2	Allied health professional	0
Women's health	4	Administrative staff	1
Child's health	4	Other	1
Other	3	Total	41
Total	41		
